# Editorial: COVID-19 pandemic and the social determinants of health

**DOI:** 10.3389/fepid.2024.1351696

**Published:** 2024-05-07

**Authors:** Rosemary M. Caron, Ronica Rooks, Mahmoud Kandeel

**Affiliations:** ^1^ School of Healthcare Leadership, MGH Institute of Health Professions, Boston, MA, United States; ^2^ Department of Health and Behavioral Sciences, College of Liberal Arts and Sciences, University of Colorado Denver, Denver, CO, United States; ^3^ Department of Biomedical Sciences, College of Veterinary Medicine, King Faisal University, Al-Ahsa, Saudi Arabia; ^4^ Department of Pharmacology, Faculty of Veterinary Medicine, Kafrelsheikh University, Kafrelsheikh, Egypt

**Keywords:** COVID-19, social determinants of health, disparity, marginalized populations, healthcare systems, health policy

## Abstract

As we learn to co-exist with COVID-19, this Research Topic highlights significant research contributions that examine the interaction of COVID-19 and the social determinants of health. To emphasize the impactful research in this area, this Research Topic features scholarly contributions in the fields of Epidemiology, specifically Aging and Life-course Epidemiology, and Public Health, specifically Public Health Policy. This theme is intentionally broad in scope, and our editorial provides an overview of the key findings of the papers published in the Research Topic on COVID-19 pandemic and the social determinants of health. The types of articles received in response to this Research Topic are summarized below.

**Editorial on the Research Topic** COVID-19 pandemic and the social determinants of health

## 1 Original research

The COVID-19 pandemic shattered the illusion of an equitable society, starkly revealing how it has deepened the gaps created by pre-existing disparities in health and socio-economic conditions across the globe. The pandemic acted as a harsh spotlight, intensifying long-existing disparities and inequities, often hidden in plain sight. Marginalized communities, particularly racial and ethnic minorities, those living in poverty, individuals with lower educational and income attainments, and those reliant on hourly wages, have been disproportionately devastated by the pandemic (1–5). This situation is a glaring reminder of the deep-rooted inequities that have been normalized or overlooked in many societies for far too long.

Syndemic research provides a vital framework for understanding the intricate and intertwined nature of socio-cultural, socio-economic, structural, and individual factors and their integrated impact on disease prevalence (6). This approach is crucial for comprehending how these determinants interact with infectious diseases like COVID-19, societal epidemics, and confinement in certain social groups. The interaction of these factors can significantly exacerbate health disparities, leading to poorer health outcomes, particularly in marginalized communities. The COVID-19 pandemic underscored the need for a comprehensive approach to public health crises that addresses these underlying social determinants of health to effectively combat and prevent future public health emergencies.

Petrelli et al. examined the difference in the incidence of intensive care (ICU), non-intensive care unit (non-ICU) hospital admissions, and mortality due to COVID-19 in the “inner areas” and metropolitan areas of Italy. The authors used a retrospective population-based study and observed a protective effect with respect to non-ICU admissions in “inner areas.” ICU admissions and mortality were also lower in these areas in the early phases of the pandemic. This protection eventually disappeared, and a slight excess risk of ICU incidence and mortality occurred during the Omicron phase of the COVID-19 pandemic. The authors proposed that the more widespread vaccination coverage in metropolitan areas may explain this observation. The authors recommended that strengthening the primary prevention policies in the surrounding Italian areas may contribute to equity in health policies.

Yin et al. examined the social factors of the COVID-19 pandemic and its evolution in Hubei, China. The authors observed regional effects of the virus based on population density, distance from the seafood market in Wuhan, China, and sufficient medical supplies. Related research conducted by Xu et al. examined the impact of COVID-19 on health services utilization in China in 2020. The authors noted a decrease in outpatient health services during this time and the reasons for this observation were multifaceted. The authors recommended that access to health services, especially emergency care, should be increased, especially during infectious disease pandemics. Meng et al. compared COVID-19 prevention and control measures between Shanghai and Beijing. The authors concluded that the social, governmental, and professional pandemic management approaches implemented should be further evaluated as different policies in these different areas were implemented and the adoption of prevention practices varied by location.

The COVID-19 pandemic tested not only medical and scientific capabilities but also highlighted the importance of psychological factors in public health. Maftei and Petroi’s study in Romania provided key insights into this often-neglected area, especially regarding vaccination behavior and the interplay between optimistic bias, conspiracy beliefs, and public perceptions. Their study became particularly relevant when considering Romania's struggle during the 2021 COVID-19 surge, which saw Europe's highest death rates and low vaccination uptake. The study highlighted the importance of psychological factors affecting public health choices, examining relationships among optimistic bias, COVID-19 conspiracy theories, vaccination status, and other behaviors like online activity and anticipated regret. A notable outcome is the strong inverse relationship between optimistic bias and the perceived threat of COVID-19, indicating that individuals who downplay their personal risk are also less likely to see the pandemic as a severe threat, thus affecting their decisions about vaccination. This research emphasized the need to focus on both the logistical and medical sides of health crises and the psychological and informational contexts in which people make health decisions. Effective public health communication should counter misinformation, tackle psychological biases, and use sophisticated approaches to reach various demographic groups, especially in a time of widespread online misinformation.

Grant and Sams examined the impact of COVID-19 lockdown measures in Africa, highlighting the limitations of a “one-size-fits-all” approach. Utilizing social media analysis, the authors investigated the diverse reactions to lockdowns across the continent, emphasizing how these measures have highlighted and exacerbated existing inequalities. Their research, grounded in social listening, examined the narratives that emerged on platforms like Twitter during the initial lockdown phase in sub-Saharan Africa. The narrative surrounding the harms of lockdowns in Africa, as captured through social media, particularly emphasized the continent's poverty and weak health systems as key risk factors for the spread of COVID-19, as well as the adverse consequences of sustained lockdown measures. The authors argued that social media became a critical space for voicing concerns and sharing knowledge, especially when traditional communication channels were disrupted by lockdowns. Grant and Sams stressed that public health responses to pandemics often failed to account for local, national, and global structural inequalities. It was suggested that social media's role in amplifying diverse voices and facilitating innovative responses to health crises, such as crowdsourcing campaigns, should be applied in future health communication strategies. The findings also advocate for the development of behavior change communication campaigns that effectively use platforms like Twitter for disseminating critical information. By acknowledging the complexity of health messaging and the contradictions inherent in epidemic response policies, policymakers can better navigate the challenges posed by health threats.

The study by López-Güell et al. evaluated the impact of COVID-19 certification mandates on case incidence and hospital admissions across the United Kingdom, revealing varied effects influenced by regional dynamics and virus variants. Certification mandates, requiring proof of vaccination, a negative test, or recent infection for public venue access, were introduced at different times across England, Northern Ireland, Scotland, and Wales. The analysis identified a decrease in cases and hospitalizations, particularly during the Delta variant's predominance. However, the study found the intervention's efficacy diminished with the emergence of the Omicron variant, especially in England, where it was less effective in reducing case incidence and hospital admissions. The discrepancy in outcomes across the UK highlighted the complex interplay between public health measures, virus variants, and population behavior. The findings suggest that while COVID-19 certification mandates contributed to increased vaccination rates and reduced transmission during the Delta variant's prevalence, their impact was less significant against Omicron. Limitations included the aggregated nature of data and potential ecological fallacy, with the study cautioning against interpreting the results as solely attributable to certification mandates due to coexisting measures and behavioral responses. The study underscores the necessity of a multi-faceted approach in pandemic management, combining certification with vaccination and other interventions tailored to evolving virus dynamics and regional contexts. It calls for continuous reassessment of public health policies to adapt to new challenges, emphasizing the importance of flexibility and evidence-based decision-making in controlling the pandemic's spread.

Kouyate et al. examined the access and use of maternal health services during the COVID-19 pandemic in Guinea. Their findings emphasize the critical need to sustain and enhance access to vital health services during a pandemic, especially for at-risk groups such as pregnant women. Initially, there was a decrease in the use of maternal health services during the early phase of the pandemic. However, some facilities later saw improvements following specific interventions including continuous training in infection prevention and control for healthcare workers, along with the distribution of delivery kits and resources during the crisis. These measures not only improved the capabilities of healthcare facilities but also boosted community trust in these services at a crucial time. The study also shed light on significant challenges, such as the inconsistent application of infection prevention strategies across various health facilities, including associated health centers, community health centers, and district hospitals. This inconsistency underscores the need for standardized health practices, especially in cleanliness and patient care protocols. Enhancing access to maternal health services during emergencies addresses immediate healthcare needs and contributes to the long-term resilience of the health system.

The COVID-19 pandemic highlighted the complex interplay between socio-structural factors and public health outcomes, as evidenced by the study conducted by Qamar et al. on COVID-19 incidence in Germany. This study sheds light on the subtle ways in which the local socio-economic environment and political opinions can greatly impact the transmission of diseases. Economic and social factors such as income, the percentage of individuals seeking protection or claiming social benefits, and the level of education seem to have minimal effect on disease occurrence rates. The association between the popularity of certain political parties and varying COVID-19 incidence suggests that public health responses and policies must consider local sociopolitical dynamics. The study advocates for a public health approach that is cognizant of these socio-behavioral factors, thereby enabling more targeted and effective interventions.

In Austria, the study by Ruf et al. examined the role of employers in influencing COVID-19 vaccine uptake among healthcare workers. Their research showed that while employers can act as influential mediators in public health decision-making, the process of choosing to vaccinate is complex and influenced by myriad factors including personal beliefs, world views, and political influences. This study sheds light on the concept of “unspoken vaccine hesitancy” among healthcare workers, emphasizing the need to create safe spaces for expressing concerns and hesitations about vaccination. The study reveals that while incentives and educational programs can increase vaccine willingness, addressing vaccine hesitancy requires a more effective approach that considers individual worldviews, political influences, and personal apprehensions. It suggests that employer-driven public health initiatives must be multi-faceted, going beyond mere information dissemination to include support systems and respect for individual decision-making processes. In doing so, it emphasized the role of employers as critical mediators in public health decision-making, especially in crisis situations like the COVID-19 pandemic.

The CRAB (COVID-19 Risk, Attitudes and Behavior) study in the Royal Navy, conducted by Woolley et al. offered insight into how knowledge, attitudes, and practices impact COVID-19 prevention. This cross-sectional analysis revealed a diverse mix of elements affecting adherence to preventive measures and reluctance to get vaccinated, emphasizing differences in how various demographic groups perceived the severity of the virus and their trust in different information sources. Key findings included lower COVID-19 seriousness ratings among male respondents and higher ratings among Black, Asian, and minority ethnic backgrounds. Among various information sources, the Defence Medical Services emerged as the most trusted for vaccine-related information. These insights are vital for understanding compliance, information credibility, and vaccine hesitancy within the Royal Navy and serve as a valuable resource for future studies on emerging infectious diseases. The research highlights the essential role of customized communication strategies in public health efforts, especially in closed, structured settings like the military.

Continuing with examining knowledge, attitudes, and perceptions, Khan et al. studied the influence of these factors on the Oxford AstraZeneca COVID-19 vaccine among primary healthcare workers in North-Central Trinidad. The main contributors to vaccine hesitancy included fear of adverse side effects, the feeling that clinical trials had not occurred for a long enough period of time, and the absence of information. Further, Fang et al. examined knowledge and attitude toward protective measures and the COVID-19 pandemic response via a questionnaire. The authors concluded that guidance should be communicated in different ways and depending on the risk presented by the health crisis, the frequency of the messaging should adapt accordingly.

The COVID-19 pandemic compelled health systems worldwide to adapt rapidly. A nationwide surveillance study in Taiwan, led by Chi et al. highlighted a significant shift in diagnostic policy during the COVID-19 epidemic from Polymerase Chain Reaction (PCR) testing to Rapid Antigen Tests (RATs). This policy change mirrored a global reevaluation of healthcare strategies in response to evolving challenges. The study underscored the vital role of RATs as a feasible, low-cost, and convenient diagnostic tool. These tests, which can be performed at home, reduced hospital visits, thereby preserving medical capacity for more severe COVID-19 cases. This work highlights the adaptive nature of health policy and its direct impact on public behavior and healthcare system strain.

Additionally, in Iran, Mohammadpour et al. conducted semi-structured interviews with healthcare experts and determined that changes were needed in several areas to respond to a future health crisis including e-health development, evidence-based decision-making, funding, collaboration at the national and international levels, and attention to the needs of healthcare workers.

## 2 Brief research report

The Mississippi Recognizing Important Vaccine & Education Resources (RIVERs) project, reported by Meador et al. emerged as a pivotal study in overcoming COVID-19 vaccine hesitancy, particularly among marginalized populations in rural or remote areas. By coordinating community engagement and local leadership, the project achieved remarkable success, notably in Mississippi, where vaccination rates among Black communities surpassed those of their White counterparts. This success underscored the critical role of local efforts in public health strategies, demonstrating how targeted interventions, grounded in the trust and influence of community leaders, can effectively combat misinformation and foster vaccine acceptance. The RIVERs project's approach, prioritizing direct community involvement and utilizing a variety of communication methods, offers a replicable model for other regions facing similar challenges. The RIVERs project faced limitations due to data aggregation at the county level, resulting in low statistical power and a cautionary note on drawing broad policy conclusions. Additionally, it lacked consideration of crucial contextual factors like vaccination access outside the program and local vaccination policies. However, the RIVERs project highlighted the importance of adapting public health initiatives to the specific needs and social contexts of vulnerable populations, ensuring that interventions are not only accessible but also resonate with the community's values and concerns.

## 3 Review

During the COVID-19 pandemic, evidence demonstrated that people of a low socioeconomic background disproportionately experienced the social and economic impacts of the COVID-19 pandemic. Nyabundi conducted a literature review to examine the roles and perceptions of social relationship networks, including kinship, as safety nets in Kenya during the pandemic. This work highlighted the need to strengthen informal familial and social support structures, which proved to be resilient during the most challenging periods of the pandemic, including addressing the socio-economic challenges brought about by COVID-19.

Lin and Wang through their systematic review, revealed how stigma, associated with the COVID-19 pandemic, is disproportionately borne by marginalized groups such as older adults, ethnic minorities, and those with lower socioeconomic status, thus underscoring the role of systemic social power imbalances. Their study advocates for a Marxist criticism approach to understand and dismantle the economic and social structures that fuel stigmatization. The mechanisms by which COVID-19 survivors faced stigmatization was through enacted stigma from communities and internalized stigma, leading to discrimination, rejection, and mental health issues. Enacted stigma included community fear and rejection, especially towards individuals in public-facing roles, while internalized stigma resulted from the survivors’ negative self-perception due to the pandemic's associated fears. This stigmatization was rooted in fear of the unknown and a lack of understanding about the virus, exacerbating social and psychological challenges for survivors. Lin and Wang call for an interdisciplinary and collective action-oriented approach. This not only aims to address and eliminate the stigma associated with health conditions like COVID-19 but also challenges us to confront and reform the underlying societal inequalities that allow such stigmas to flourish. Their work serves as a critical reminder of the importance of looking beyond individual attributes to the systemic forces at play in exacerbating social inequality and stigmatization, urging for a comprehensive renovation of our social care systems to ensure a more equitable society.

## 4 Policy and practice reviews

Despite the documented success of many public health policies (e.g., smoking bans), Humphries et al. state that there is a need for values and varied perspectives to be considered during the policy analysis process. In particular, the authors implemented the Intersectionality-based Policy Analysis (IBPA) framework, which examines problems and policy approaches via a guiding principles approach. The authors applied the IBPA framework to the COVID-19 pandemic specifically to examine racial conflict and resolution in the United States of America via a participatory approach that utilized reflection and open-ended questions. The authors report that the tool was useful in identifying problems or policies and their respective impacts on different population groups.

## 5 Opinion

Chatelan and Khalatbari-Soltani’s commentary serves as a call for transforming the traditional approach in public health of targeting high-risk individuals through specific interventions. The authors stated that this method falls short in addressing the continuing health disparities seen in socially vulnerable groups, such as racial and ethnic minorities, or those of a lower socioeconomic status. The authors suggest adopting a dual strategy that not only focuses on interventions aimed at the general population but also gives special attention to programs for these vulnerable communities. The COVID-19 pandemic has highlighted the limitations of solely focusing on personal responsibility and broad population measures. Future public health interventions must be centered around the needs of the population and the social determinants of health that impact health outcomes.

## 6 Perspective

The study by Mortimer et al. is a reminder of the general impact of racism on public health. The authors remind us that as we navigate to the post COVID-19 pandemic, it is imperative that efforts to combat racism and related social determinants are placed at the forefront of public health strategy. The COVID-19 pandemic unmasked deep-rooted structural issues in public health, with racism emerging as a critical determinant impacting health outcomes and demonstrating how the pandemic exacerbated existing disparities and disproportionately affected racial and ethnic minorities. Factors such as residential segregation, economic insecurity, and discrimination have long been shaping the health outcomes of minority populations. The pandemic heightened the visibility of these pre-existing conditions and provided a unique opportunity to re-think and reform our approach to public health.

## 7 Conclusion

The research highlighted herein demonstrates research contributions from a unique time in our history where we had to respond and prevent a complex, multi-factorial health threat that disproportionately impacted the most vulnerable among us. The work that comprises the Research Topic, COVID-19 Pandemic and the Social Determinants of Health, represents important and impactful recommendations for how we should prepare for ongoing and future global health threats.
